# Special Issue: Pathogenic *Escherichia coli*: Infections and Therapies

**DOI:** 10.3390/antibiotics10020112

**Published:** 2021-01-25

**Authors:** Margarita Martinez-Medina

**Affiliations:** Microbiology of the Intestinal Disease, Biology Department, University of Girona, 17003 Girona, Spain; marga.martinez@udg.edu

*Escherichia coli* is a facultative anaerobic Gram-negative bacterium from the Enterobacteriaceae family that colonizes the gastrointestinal tract of warm-blooded animals shortly after birth, and it is a lifelong colonizer of adults. This species persists as a harmless commensal in the mucous layer, interacting with the host in a mutualistic manner. However, there are certain strains with pathogenic properties that can cause disease, and, in turn, nonpathogenic intestinal *E. coli* can eventually cause or contribute to disease in compromised hosts. 

Pathogenic *E. coli* strains are grouped into pathotypes according to their clinical spectra and virulence factors. The extraintestinal pathogenic *E. coli* (ExPEC) group comprises the strains causing infections outside the intestinal tract, mainly infections in the urinary tract, but also sepsis, meningitis, and wound infections. These strains are genetically diverse and resemble nonpathogenic *E. coli* residing in the intestinal tract. Distinct from commensal and ExPEC strains, diarrhoeagenic *E. coli* (DEC) cause intestinal infections and harbor specific surface adhesins and other virulence factors, and they can be classified into seven well-defined pathotypes: enterohemorrhagic *E. coli* (EAEC), enteropathogenic *E. coli* (EPEC), enterotoxigenic *E. coli* (ETEC), enteroaggregative *E. coli* (EHEC), enteroinvasive *E. coli* (EIEC), diffusely adherent *E. coli* (DAEC), and necrotoxic *E. coli* (NTEC). Recently, a new pathotype called adherent-invasive *E. coli* (AIEC) has been proposed as being associated with inflammatory bowel disease, especially Crohn’s disease.

As Gram-negative organisms, *E. coli* are resistant to many antibiotics, and high-risk *E. coli* multiresistant clones are emerging. Indeed, carbapenem-resistant extended-spectrum beta-lactamase (ESBL)-producing strains are considered to be of critical priority by the World Health Organization as bacterial pathogens for which new antibiotics should be designed. On the other hand, antibiotic treatments have profound effects on the human microbiome, and, thus, new strategies, such as very-narrow-spectrum treatments, antiadhesives, phage therapy, or vaccination, are welcomed.

This Special Issue “Pathogenic *Escherichia coli*: Infections and Therapies” consists of 12 articles, including original research on intestinal pathologies and extraintestinal infections of human and animal origins caused by *E. coli* [1,2,3,4,5,6,7] and a review on FimH antiadhesive molecules that can be used to treat urinary tract infections caused by uropathogenic *E. coli* (UPEC) [8]. Apart from this review, other original articles propose novel approaches to treat and/or prevent *E. coli* infections. For instance, Zhang et al. suggest that colonic butyrate administration could be a novel treatment approach to decrease the growth and virulence gene expression of dysbiotic pathosimbiont *E. coli* in the context of inflammatory bowel disease [9]. Bumunang et al. propose bacteriophage therapy and phlorotannin as antimicrobials against biofilm-forming Shiga toxin-producing *E. coli* [10], and Alves et al. propose poly(MeOEGMA) as a polymer brush that prevents bacterial adhesion in urinary tract devices, such as ureteral stents and catheters, which has the potential to eradicate biofilms developed in these biomedical devices [11]. On the other hand, Mazurek-Popczyk et al. identify bacteriocin-producing *E. coli* in the human intestine and demonstrate that these strains have antibacterial activity against zoonotic *E. coli*, thus demonstrating the importance of these strains in hampering the colonization of the human intestine by animal strains and, therefore, in preventing zoonotic infections [12].

The focus of the Special Issue is rather wide; nonetheless, we do expect that this group of manuscripts will be of interest to the research community interested in pathogenic *E. coli* virulence properties, antimicrobial resistance mechanisms, transmission paths, and novel potential therapies and prevention approaches.
